# Towards Targeted Interventions in Low- and Middle-Income Countries: Risk Profiles of People Who Inject Drugs in Haiphong (Vietnam)

**DOI:** 10.1155/2020/8037193

**Published:** 2020-09-10

**Authors:** Adeline Riondel, Duong Thi Huong, Laurent Michel, Marianne Peries, Khuat Thi Hai Oanh, Pham Minh Khue, Nham Thi Tuyet Thanh, Hoang Thi Giang, Roselyne Vallo, Amandine Cournil, Delphine Rapoud, Catherine Quillet, Didier Laureillard, Vu Hai Vinh, Jean-Pierre Moles, Jonathan Feelemyer, Ted Hammett, Don Des Jarlais, Nicolas Nagot

**Affiliations:** ^1^Pathogenesis and Control of Chronic Infections, University of Montpellier, Inserm U1058, Etablissement Français du Sang, Montpellier University Hospital, 60 Rue de Navacelles, 34394 Montpellier, France; ^2^Hai Phong University of Medicine and Pharmacy, 72A Nguyen Binh Khiem, 18000 Hai Phong City, Vietnam; ^3^CESP/Inserm U1018, Paris-Sud University and Paris Descartes University, Centre Pierre Nicole, French Red Cross, 27 Rue Pierre Nicole, 75005 Paris, France; ^4^Supporting Community Development Initiatives, 240 Mai Anh Tuan, Cho Dua, Dong Da, Hanoi, Vietnam; ^5^Infectious Diseases Department, Caremeau University Hospital, Place du Pr R. Debré, 30029 Nîmes, France; ^6^Department of Infectious and Tropical Diseases, Viet Tiep Hospital, Hai Ba Trung, Cat Dai, Hai Phong, Vietnam; ^7^College of Global Public Health, New York University, New York, USA; ^8^Abt Associates, 55 Wheeler Street, 02138 Cambridge, Massachusetts, USA

## Abstract

People who inject drugs (PWID) are a dominant risk group afflicted by blood-borne viruses, mental health disorders, and social precariousness. Risk reduction interventions are administered to PWID regardless of their characteristics or specific risks. The objective of this cross-sectional analysis was to empirically identify profiles of PWID regarding their drug use, risk behaviors, and mental health in order to tailor adapted interventions taking into account limited access to comprehensive care in middle-income countries. PWID were recruited using respondent-driven sampling. PWID with urine testing positive for heroin or methamphetamine and manifesting recent skin injection marks were enrolled. Classification of participants was based on drug use, injection, risky sexual behavior, and mental health data. This was subjected to multiple correspondence analysis followed by hierarchical cluster analysis combined with *K*-means methodology. From October 2016 to January 2017, 1490 participants were recruited of which 1383 were eligible and enrolled. HCV prevalence was 70.5% and HIV prevalence 29.4%. The cluster analysis identified five distinct profiles: profile 1: recent injection practices and high alcohol consumption, profile 2: at-risk injection and sexual behaviors with precarious situations, profile 3: no sexual activity and older age, profile 4: frequent injections with high methamphetamine use, and profile 5: stable partnerships and less frequent injections. Our study has identified profiles of PWID at particularly high risks, and they should thus be targeted for interventions tailored to their specific risks.

## 1. Introduction

Vietnam is particularly concerned with illicit drug use since the economic reform promoted liberalization of the economic market in 1986 (Doi Moi) [1]. Drug supply was also facilitated by its close proximity to the Golden Triangle [2]. There were approximately 200,000 people who inject drugs (PWID) in 2012 in Vietnam [3]. Since 1993, the political response to drug use was to send PWID to rehabilitation centers without any addiction care [4]. Several preventive or medical interventions have been implemented since the early 2000s. In 2002, needle/syringe exchange programs were expanded throughout Vietnam for HIV control, reducing new HIV infections by 23% between 2002 and 2009 [5]. Subsequently, in 2008, Haiphong pioneered methadone clinics [5]; there were 13 in the city in 2017. In contrast, HCV has been neglected in Vietnam. HCV prevalence in 2014 was approximately 74% among PWID in Vietnam [6] and approximately 67% in Haiphong [7]. We recently estimated both the incidences of HIV and HCV infections among PWID in Haiphong; we reported a low HIV incidence (between 0 and 1.8 per 100 person-years (PY), contrasting with a very high HCV prevalence (19.4/100 PY, 95% CI: 11.5-30.7) [8].

Mental health disorders among PWID are often associated with the severity of substance use disorder and higher risk practices [9–11]. Nevertheless, access to mental health care remains very limited in Vietnam, particularly for PWID. However, promising mental health community-based interventions have been described elsewhere, including group interpersonal psychotherapy, counseling sessions at home, and community reentry programs [12–14]. As in many countries, these PWID interventions are copied from those elaborated in other settings and delivered according to a “one-fits-all” approach. This may not address the wide diversity of PWID [15] as well as the different settings, particularly in middle-income countries where access to specialized care (addiction treatment, psychiatric care, and harm reduction settings) is limited. HCV control mainly based on harm reduction towards safer injection practices stands as a good example, but this concern also applies to all other interventions implemented for PWID. Since these interventions are mainly led by peers within small groups of PWID, an approach tailored to the specific risks may make a difference [16].

In order to have information on risk reduction activities, knowledge of the different profiles of PWID is crucial. The objective of this study was therefore to identify and describe the various profiles of PWID regarding their drug use, risk behaviors, and mental health in order to tailor adapted community-based interventions in Haiphong, Vietnam.

## 2. Materials and Methods

### 2.1. Study Design

We conducted a secondary analysis of data collected for a cross-sectional respondent-driven sampling (RDS) survey within the interventional Drugs & Infections in ViEtnam (DRIVE) research project. RDS, first described by Heckathorn, is the most suitable method for recruiting a representative sample of hard-to-reach populations [17–19].

### 2.2. Settings

The 2016 RDS survey was implemented in Haiphong City (approximately two million inhabitants) with active involvement in four Community-Based Organizations (CBO) in several aspects of the study. The RDS survey took place between October 2016 and January 2017 in two CBO offices (Light House and Friendship Arms).

The research protocol was approved by the institutional review boards of Haiphong Medical University, Hanoi Medical University, and Icahn School of Medicine (New York City). It is registered under www.clinicaltrials.gov (NCT03526939).

### 2.3. Participants and Study Conduct

Given that our target population was PWID at high risk (i.e., those still frequently injecting), we recruited individuals aged 18 years and above with urine testing positive for heroin or methamphetamine and manifesting recent skin injection marks (ascertained by CBO members). Only eligible PWID having signed an informed consent form after being informed about the study were enrolled.

Twenty seeds (10 individuals from each site) were selected to initiate the RDS. These included PWID recently, PWID for a long time, sex workers who inject drugs, and men who have sex with men who inject drugs from various districts. As the proportion of women among PWID was expected to be low in Haiphong (5%), we set the male/female seed ratio at 4/1 to ensure their inclusion. The seeds received three coupons each on the first day of RDS and were told by the CBO members to give the coupons to other PWID actively injecting and within their network. Each coupon was valid for one week. The network size of each participant was collected in the RDS Coupon Manager® 3.0 software.

One month after recruitment, the number of participants recruited per day decreased below the initial target. This was mainly due to police harassment at “hotspots” where PWID were known to congregate and purchase drugs. In order to improve cohort size, we added five new seeds and temporarily increased the number of coupons up to 10 for the very few participants describing a very large network. This strategy was actually not very successful and was discontinued, with only 33 participants recruiting more than four other PWID.

Coupon validity was extended to two weeks. Participants were compensated VND 150,000 (approximately US $7.50), plus VND 50,000 (approximately USD $2.50) for each PWID they successfully recruited. They also received an additional VND 50,000 for travel to and from the study site for HIV and HCV test results.

### 2.4. Data Sources and Measurements

Urinary tests were completed using Drug-screen Multi 7A card (Nal von Minden, Moers, Germany) and read by the BLOTrix C1 reader piloted by B4C software (BioSciTec, Frankfurt, Germany). The detection threshold was set at 300 ng/mL for heroin and 500 ng/mL for methamphetamine. A fingerprint machine allowed unique identification of participants and avoided repeated enrollments.

HIV and HCV serology was assessed during the study visit at the sites. HCV serology testing was performed using the SD Bioline HCV rapid test (Standard Diagnostics Inc., Gyeonggi-do, Republic of Korea) and HIV serology testing with the Determine™ HIV-1/2 (Alere™, Waltham, USA) rapid test. Confirmation of HIV detection was carried out using the Bioline HIV1/2 3.0 rapid test (Standard Diagnostics Inc., Gyeonggi-do, Republic of Korea) plus the VIKIA® HIV1/2 test (Marcy-l'Etoile, Lyon, France) at the provincial AIDS referral laboratory on blood samples collected during the study visit.

A standardized questionnaire was administered by a trained research assistant to collect information on demographic characteristics, drug use with injection practices, sexual behaviors, contact with drug treatment services, and medical history. The 4-item Patient Health Questionnaire (PHQ4) was used to screen for anxiety and depression [20]. Drug use and sexual behaviors were documented for the prior six months.

Alcohol consumption was assessed by using the AUDIT-C score [21]. At-risk alcohol consumption was defined as a score of 4 and above for men and 3 and above for women. Binge drinking was defined as consumption of 5 or more drinks on one occasion at least once per month.

We defined unsafe sex when a condom was not always used (i) during sexual intercourse with a primary partner with unknown or positive HIV/HCV status and (ii) during sexual intercourse with a casual partner.

All data were entered into an eCRF complying with FDA requirements (21 CRF part 11).

### 2.5. Statistical Methods

The number of waves required to reach equilibrium, the homophily of the main variables, and their crude and adjusted estimates were calculated with RDSAT® (version 7.1.46). A number of waves of less than six and a homophily around zero for key parameters, such as HCV or HIV serostatus, were used to assess the RDS validity. All seeds were included in the analysis.

We used a cluster analysis to determine different profiles according to their risk behaviors towards blood-borne viruses, drug/alcohol use, sexual behavior, and mental health status. We first conducted a multiple correspondence analysis (MCA) to reduce the dimensions of the data set using active variables reflecting drug use, risk behaviors, and mental health (Table 1). Briefly, active variables included items related to heroin and other drug use, injection and risky sexual behaviors, methadone use, and mental health. We did not include HCV or HIV status among the active variables as they reflected former risk factors. The number of dimensions/axes maintained was determined using the elbow criteria. The Benzecri adjustment was applied to calculate the contribution of the selected axes to the total inertia [22]. The MCA output was then used to perform the cluster analysis in order to identify subgroups (clusters) of participants with specific risk profiles. The clustering was based on a mixed method combining a hierarchical cluster analysis (HCA), to determine the optimal number of subgroups, and the *K*-means method, to define the clusters by assigning each participant to one of the subgroups [23] (see supplementary data, Table S2 and Figures S1 and S2).

The profiles were then characterized by describing the distribution of active variables, as well as other variables that were not used for the cluster analysis, such as sociodemographic variables. The stability of the classification was checked by modifying the number of axes of the MCA.

Statistical analyses were conducted using SAS® version 9.4 (SAS Institute Inc., Cary, USA).

## 3. Results

### 3.1. Participants

**Figure 1:**
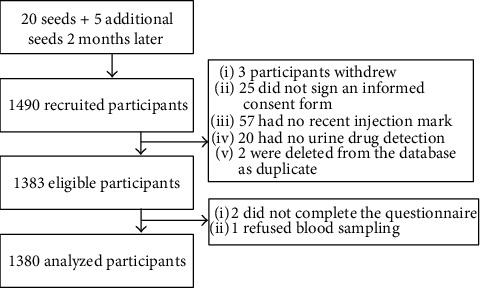
Participant workflow.

**Figure 2:**
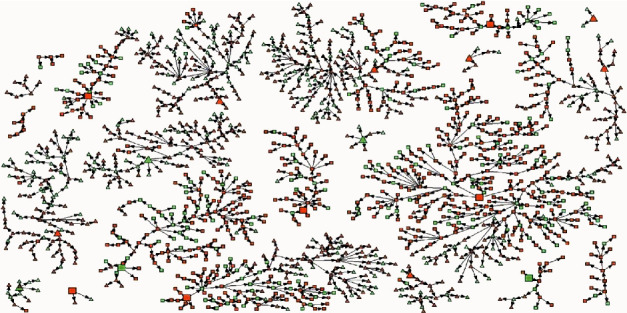
RDS recruitment network according to HCV serostatus. Squares and triangles represent participants who were recruited at Friendship Arms office or Light House office, respectively. HCV-seropositive participants are shown in orange and seronegative ones in green. Large data points represent the seeds.

Among the 5075 coupons issued, 1490 coupons were returned (29.4%). Each seed recruited between zero and three participants, and there were 16 waves of recruitment. We encountered some challenges during participant recruitment. These included a few attempts of duplicated participation detected by the fingerprint device, enhanced police enforcement limiting possibilities for PWID to meet in public areas/hotspots and distribute coupons, and finally a typhoon which interrupted the study process for two days. In all, 1380 participants were enrolled and analyzed. The participant flowchart is presented in Figure 1 and the recruitment network in Figure 2.

The equilibrium was reached at the 1^st^ wave for HCV, the 2^nd^ wave for HIV, and the 3^rd^ wave for gender. The homophily was 0.02 for HCV+, 0.1 for HIV+, and 0.1 for gender, thus meaning that the recruitment was performed randomly and not according to participant characteristics.

The unweighted prevalence was 29.8% (27.4-32.3) for HIV and 70.4% (67.9-72.8) for HCV. After weighting with the RDSAT® estimator, the prevalence remained very similar (Supplementary Table S2). Therefore, we conducted further analyses considering samples as a representative of the population of interest without accounting for the design effect.

Participants had a mean age of 39 years and were predominantly male (93.8%). All participants injected heroin, and some (*n* = 23, 1.7%) also injected methamphetamine. The participant characteristics are shown in Table 1.

### 3.2. Determination of PWID Profiles

From the MCA, we selected seven axes which accounted for approximately 70% of the total inertia (Supplementary Table S2). They were defined as follows: sexual activity, risky injection and sexual behavior, injection frequency and methadone use, alcohol consumption, drug use, duration of injection, and overdose.

We identified five subgroups from the HCA and used them to carry out the *K*-means analyses.

We describe the population profiles as follows (Table 2):

#### 3.2.1. Profile 1: Recent Injection Practices and High Alcohol Consumption (*N* = 176)

This profile consists of PWID who started to inject more recently but injected less frequently than other profiles. They were predominantly males, and all presented hazardous alcohol consumption, such as frequent binge drinking.

#### 3.2.2. Profile 2: At-Risk Injection and Sexual Behaviors with Precarious Situations (*N* = 79)

PWID from this profile tended to inject with an already used syringe, shared water, and had the highest overdose rate. They reported risky sexual behaviors with primary or casual partners more frequently than the other profiles. The psychological assessment score reflected a probable mental health disorder. They had the most precarious situations. Indeed, they were the youngest PWID with the most illegal sources of income, the highest proportion of homelessness, and lack of ID card.

#### 3.2.3. Profile 3: Older Age without Sexual Activity (*N* = 467)

These PWID were the oldest, being more frequently single and without sexual activity. They injected heroin safely and moderately every day, with less use of methamphetamine and methadone.

#### 3.2.4. Profile 4: Frequent Injections with Safe Behaviors but High Methamphetamine Consumption (*N* = 317)

These PWID had the highest frequency of injection (three-four times a day). This profile had the highest proportion of women, methamphetamine uses, and other drug uses. These PWID did however practice safe injection and had safe sexual behaviors.

#### 3.2.5. Profile 5: Stable Partnerships and Less Frequent Injections (*N* = 341)

These PWID injected less than those in the other profiles. They used less methamphetamine, and they exhibited less sexual and injection risk behaviors. They had the highest level of education and lived in partnerships with almost no casual partners.

### 3.3. Sensitivity Analyses

Varying the number of axes before HCA (up to 12) and the number of classes selected for the *K*-means (from four to six) did not improve the characterization of the profiles (data not shown).

## 4. Discussion

We identified five profiles of PWID in Haiphong City exhibiting different levels of drug use, drug- or sex-related risk behaviors, and mental health disorders. Among the profiles, three (profiles 1, 2, and 4) demonstrate behaviors commonly associated with transmission of blood-borne viruses, either through unsafe injection practices, unsafe sex, high stimulant (methamphetamine) consumption, or at-risk alcohol consumption. These three profiles, representing 41% of PWID, are priority targets for blood-borne virus control interventions. Profile 2 could particularly expose PWID to HCV transmission through frequent water/novocaine sharing (70%) in addition to syringe sharing. It should be noted that this latter group reported the lowest rate of HCV awareness and testing. Furthermore, these PWID were the youngest and the most at risk with respect to several aspects (social precariousness, former history of overdosing, mental health condition, and at-risk sexual behavior). They also reported the highest rate of street methadone use. CBO members indicated that the use of street methadone was a way to cope with heroin shortage. Outreach interventions targeting awareness on transmission of blood-borne viruses, rapid on-site testing, free injection solution provisions, and facilitation of free access to methadone treatment would likely be adapted to this subpopulation.

PWID in profile 1 were all at-risk drinkers. Although it was not demonstrated by our data, possibly due to underreporting, alcohol use is known to be associated with disinhibited behaviors and risk-taking practices [24, 25]. Alcohol use should be systematically assessed among PWID, and brief intervention should be offered to all at-risk drinkers [26]. PWID in profile 4 were mainly characterized by methamphetamine use (96%) which is also associated with sex- or drug-related risk practices. Nevertheless, in our study, very few PWID were injecting methamphetamines; it was almost exclusively smoked. Indeed, risks could then rather be related to inappropriate practices when injecting heroin during methamphetamine rush, unsafe sex, and also HCV transmission when sharing pipes with lip burns. These PWID were more often female, injecting more frequently, and more often polydrug users. Most women who inject drugs are sex workers, and CBO members reported that polydrug use with clients was a common practice among sex workers. These findings are consistent with a study conducted in Tijuana, Mexico, where stimulant smoking was associated with polydrug use and transactional sex [15]. In contrast with our study, highlighting that unsafe sex was not frequent in this profile, it was associated with high-risk sexual behavior in the Mexican setting. Interventions should probably target information on methamphetamine use, prevention of psychiatric disorders related to methamphetamine use, and prevention of transition to methamphetamine injection. The other two profiles (profiles 3 and 5) exhibit lower risk profiles. It should be noticed that among PWID in profile 5, very few reported sexual intercourse with casual partners, but they had the highest rate of unprotected sex with their primary partner. Screening and awareness for the primary partner should be proposed.

Our study presents some limitations. We cannot exclude a selection bias due to police enforcement that changed the drug scene, reducing contacts between PWID due to the elimination of many drug purchasing hotspots. Therefore, the most precarious PWID may be underrepresented in this study. A desirability bias is always possible during a face-to-face interview. Indeed, questions on injection practice, drug use, and sexual behaviors are very sensitive as they are designed to elicit very personal information. Some participants may have underreported certain behaviors because they felt embarrassed or guilty regarding these behaviors. Finally, some HCV infection risk factors were not evaluated, including tattoos, blood transfusion, or surgery history [27, 28].

One of the strengths of our study is that it describes a hard-to-reach population having never before been characterized in terms of risk profiles. We were able to enroll a large sample size, about the third of the total population of active PWID, likely being representative of the target population, given that the RDS assumptions were met [29]. Finally, we adopted a cluster analysis which is likely the most appropriate approach to define various profiles of a marginalized population [30]. Our study also showed different PWID profiles and the persistence of risk behaviors among some PWID.

Prevention campaigns and needle/syringe exchange programs are efficient in decreasing both HIV and HCV. However, in this setting, additional interventions are required to tackle HCV transmission, which remains highly prevalent and incident. In our study, very few participants reported sharing needles and syringes (around 5%). HCV routes of transmission are not well known by PWID, in particular transmission via water or other dilution liquids, i.e., novocaine, and very few PWID were tested for HCV despite their high level of risk. These practices should be targeted by preventive interventions. The risk of HCV transmission with methamphetamine use is also possible. Indeed, formation of microlesions by lip burns during smoking can transmit HCV if smoking material is shared [31]. Again, these risk behaviors should be targeted by dedicated prevention activities, such as the availability of safer methamphetamine kits which include glass stems and rubber mouthpieces [32]. Focusing prevention on sharing drug use material and a good knowledge of the disease are necessary to effectively manage and control HCV spread among PWID. Our findings suggest that resource-limited HCV control should first target the profiles of PWID with a high potential of HCV transmission. During peer-led interventions, a quick assessment could be done to identify to which profile the individual belongs in order to then deliver interventions tailored to his or her profile-specific risks. For low-risk profiles, minimal general information on HCV infection could be provided, but the risk level should be maintained over time through continuous counseling.

In addition to primary and secondary prevention, new treatments for HCV with direct action could reduce HCV epidemics among PWID if used with high coverage. This would be via reducing the HCV viral load at the population level, i.e., the number of individuals transmitting the virus. Although the cost of these treatments was prohibitive until recently, several initiatives are underway to open their access in resource-limited countries.

Our findings cannot be generalized to all PWID. We decided to focus on the PWID most at risk of transmission of blood-borne viruses by obtaining evidence of heroin injection (urine testing and recent skin injection marks) which excluded individuals carrying out recreational injections and/or those successfully controlled on methadone and thus at low risk of HCV transmission. Therefore, our sample typically consisted of PWID “from the street.” These people inject drugs frequently and have no or little contact with the health care system but on the other hand could benefit from harm reduction activities. Although we had to marginally modify some of the RDS rules, the decent representativeness of our sample led us to obtain robust estimations of the target population. Our results could therefore be of interest for other urban settings in Asia.

## 5. Conclusion

Our study showed that the PWID population in large cities, such as Haiphong, is made of distinct profiles of individuals with varying levels of infection risk. Given that resources are limited, particularly the availability of peers, the knowledge of these profiles is crucial to target those with the highest risk and to subsequently tailor their interventions.

## Figures and Tables

**Table 1:** Drug use, risk behaviors, and mental health characteristics (active variables) in study participants.

	*N*	%	95% CI
Number of years since the first injection of heroin			
<5	396	28.7	26.3-31.2
5 to <10	357	25.8	23.6-28.2
10 to <15	296	21.4	19.3-23.7
≥15	331	24.0	21.8-26.4
Number of days of heroin injection, last month			
≤15	111	8.0	6.7-9.6
>15	161	11.7	10.4-13.5
Everyday	1108	80.2	78.9-83.5
Number of heroin injections in a typical day, last month			
1	235	17.0	15.1-19.1
2	616	44.6	42.0-47.3
3	439	31.7	29.3-34.3
≥4	90	6.5	5.3-8.0
Smoked methamphetamine use			
Never	386	28.0	25.6-30.4
Ever used but not in the last month	321	23.3	22.8-25.7
Used in the last month	673	48.8	46.2-51.4
Other drug use^a,b^			
Cannabis only	247	17.9	15.8-19.9
Other^c^	442	32.1	30.1-35.1
At-risk alcohol consumption^a^	421	30.4	28.0-33.1
Binge drinking^a^	168	12.2	10.4-14.2
Use of street methadone^a^	533	38.6	37.3-40.5
Currently receiving prescribed methadone	164	11.8	10.2-13.7
Overdose associated with a loss of consciousness^a^	47	3.4	2.5-4.5
Use of a shared needle/syringe^a^	68	4.9	3.8-6.0
Use of shared water/novocain^a^	187	13.6	11.8-15.5
Safe sex with a primary partner^a^	495	35.8	33.3-38.4
Unsafe sex with a primary partner^a^	123	8.9	7.4-10.5
Safe sex with a casual partner^a^	155	11.2	9.6-13.0
Unsafe sex with a casual partner^a^	82	5.9	4.8-7.4
Depression and anxiety (PHQ4)			
None	979	70.9	68.5-73.3
Mild	298	21.6	19.5-23.9
Moderate	69	4.9	3.9-6.3
Severe	34	2.4	1.7-3.4

^a^In the past 6 months; ^b^in addition to heroin and methamphetamine; ^c^among amphetamine, benzodiazepine, cocaine, methadone, and cannabis.

**Table 2:** Profiles' characteristics.

	Profile 1	Profile 2	Profile 3	Profile 4	Profile 5
	*N* = 176	*N* = 79	*N* = 467	*N* = 317	*N* = 341
	(12.8%)	(5.7%)	(33.8%)	(23.0%)	(24.7%)
Gender (male/transgender)	174 (98.9)	73 (92.4)	445 (95.3)	288 (90.9)	316 (92.7)
Age < 39 years old	85 (48.3)	51 (64.6)	218 (46.7)	159 (50.2)	178 (52.2)
Grade of school: middle/high, university	124 (70.5)	56 (70.9)	326 (69.8)	219 (69.1)	256 (75.1)
Illegal sources of income	25 (14.2)	22 (27.9)	117 (25.1)	76 (24.0)	56 (16.4)
Single, widowed, divorced	174 (98.9)	73 (92.4)	445 (95.3)	288 (90.9)	316 (92.7)
Homelessness	10 (5.7)	7 (8.9)	29 (6.2)	18 (5.7)	9 (2.6)
Lack of ID card	54 (30.7)	31 (39.3)	167 (35.8)	112 (35.3)	87 (25.5)
Number of years since the first injection of heroin					
<5	66 (37.5)	24 (30.4)	113 (24.2)	90 (28.4)	103 (30.2)
5 to <10	38 (21.6)	20 (25.3)	133 (28.5)	83 (26.2)	83 (24.3)
10 to <15	39 (22.2)	14 (17.7)	105 (22.5)	73 (23)	65 (19.1)
≥15	33 (18.8)	21 (26.6)	116 (24.8)	71 (22.4)	90 (26.4)
Number of days of heroin injection, last month					
≤15	24 (13.6)	4 (5.1)	32 (6.9)	27 (8.5)	24 (7)
>15	26 (14.8)	10 (12.7)	42 (9)	32 (10.1)	51 (15)
Everyday	126 (71.6)	65 (82.3)	393 (84.2)	258 (81.4)	266 (78)
Number of heroin injections in a typical day, last month					
1	22 (12.5)	11 (13.9)	90 (19.3)	36 (11.4)	76 (22.3)
2	91 (51.7)	35 (44.3)	211 (45.2)	126 (39.8)	153 (44.9)
3	55 (31.3)	28 (35.4)	140 (30)	119 (37.5)	97 (28.5)
≥4	8 (4.6)	5 (6.3)	26 (5.6)	36 (11.4)	15 (4.4)
Smoked methamphetamine use					
Never	35 (19.9)	6 (7.6)	206 (44.1)	7 (2.2)	132 (38.7)
Ever used but not in the last month	34 (19.3)	16 (20.3)	141 (30.2)	6 (1.9)	124 (36.4)
Used in the last month	107 (60.8)	57 (72.2)	120 (25.7)	304 (95.9)	85 (24.9)
Other drug use^a,b^					
Cannabis only	28 (15.9)	12 (15.2)	125 (26.8)	7 (2.2)	75 (22)
Other^c^	60 (34.1)	35 (44)	24 (5.1)	297 (93.7)	27 (7.9)
At-risk alcohol consumption^a^	176 (100)	55 (69.6)	393 (84.2)	252 (79.5)	258 (75.7)
Binge drinking^a^	159 (90.3)	9 (11.4)	1 (0.2)	0	0
Use of street methadone^a^	74 (42.1)	42 (53.2)	151 (32.3)	123 (38.8)	143 (41.9)
Currently receiving prescribed methadone	14 (8)	6 (7.6)	57 (12.2)	45 (14.2)	42 (12.3)
Overdose associated with a loss of consciousness^a^	11 (6.3)	9 (11.4)	15 (3.2)	3 (1)	9 (2.6)
Use of a shared needle/syringe^a^	1 (0.6)	61 (77.2)	2 (0.4)	1 (0.3)	3 (0.9)
Use of shared water/novocain^a^	17 (9.7)	55 (69.6)	56 (12)	25 (7.9)	34 (10)
Unsafe sex with a primary partner^a^	25 (14.2)	11 (13.9)	0	28 (8.8)	59 (17.3)
Unsafe sex with a casual partner^a^	24 (13.6)	12 (15.2)	15 (3.2)	28 (8.8)	0
Depression and anxiety (PHQ4)					
None	129 (73.3)	25 (31.7)	363 (77.7)	204 (64.4)	258 (75.7)
Mild	31 (17.6)	41 (51.9)	76 (16.3)	79 (24.9)	71 (20.8)
Moderate	12 (6.8)	10 (12.7)	23 (4.9)	18 (5.7)	6 (1.8)
Severe	4 (2.3)	3 (3.8)	5 (1.1)	16 (5.1)	6 (1.8)
Ever heard of HCV	110 (62.9)	45 (56.4)	279 (60.1)	205 (65.9)	211 (62.2)
Ever tested for HCV	39 (22.3)	10 (12.8)	116 (25.0)	82 (26.4)	82 (24.3)

^a^In the past 6 months; ^b^in addition to heroin and methamphetamine; ^c^among amphetamine, benzodiazepine, cocaine, methadone, and cannabis; values are numbers and percentages (enclosed in parentheses).

## Data Availability

The full data set is available upon request to NN.

## Supplementary Materials

Supplementary Materials Table S1 provides a description of weighted and unweighted estimates of the main characteristics of the study population. Table S2 and Figures S1 and S2 provide additional information on the cluster analysis.
