# Supplementary material for: Towards Targeted Interventions in Low- and Middle-Income Countries: Risk Profiles of People Who Inject Drugs in Haiphong (Vietnam)
Source: Biomed Res Int. 2020 Sep 10;2020:8037193. doi: 10.1155/2020/8037193 (PMC7502134; doi:10.1155/2020/8037193)
Supplement: Supplementary Materials — Table S1 provides a description of weighted and unweighted estimates of the main characteristics of the study population. Table S2 and Figures S1 and S2 provide additional information on the cluster analysis. [file 8037193.f1.docx]

Table S1. Population characteristics with unweighted and weighted estimates

|  | % Unweighted [95% CI] | | % Weighted [95% CI] |
| --- | --- | --- | --- |
| Gender |  | |  |
| Female | 6.1 [4.9 - 7.5] | | 5.7 [4.3 - 7.4] |
| Male | 93.7 [92.3 - 95.0] | | 94.1 [92.3 - 95.5] |
| Transgender | 0.2 [0.06 - 0.7] | | 0.2 [0 - 0.7] |
| Marital status |  | |  |
| Single | 34.9 [32.4 - 37.5] | | 34.4 [31.2 - 37.4] |
| Legally married | 33.4 [31.0 - 36.0] | | 34.4 [31.3 - 37.7] |
| Living in couple | 3.2 [2.4 - 4.4] | | 2.5 [1.5 - 3.3] |
| Divorced/Separated | 27.2 [24.9 - 29.7] | | 27.7 [25 - 30.8] |
| Widowed | 1.2 [0.7 - 2.0] | | 1.2 [0.5 - 1.8] |
| Years of injection | |  |  |
| <5 | 28.7 [26.4 - 31.2] | | 29.4 [26.4 - 32.2] |
| 5 to <10 | 25.8 [23.5 - 28.2] | | 26.7 [24 - 29.7] |
| 10 to <15 | 21.4 [19.3 - 23.7] | | 19.8 [17.5 - 22.3] |
| ≥ 15 | 24.0 [21.8 - 26.4] | | 24 [21.2 - 26.9] |
| HIV |  | |  |
| HIV positive serology | 29.8 [27.4 - 32.3] | | 29.4 [26.4 - 32.8] |
| HCV |  | |  |
| HCV positive serology | 70.4 [67.9 - 72.8] | | 70.5 [67.7 - 73.4] |

Note. Respondent-driven sampling (RDS), combines "snowball sampling" (getting individuals to refer those they know, these individuals in turn refer those they know and so on) with a mathematical model that weights the sample to compensate for the fact that the sample was collected in a non-random way”. In our study, unweighted and weighted estimates shown in table S1 were very similar. We therefore conducted analyses considering the sample as representative of the population of interest and without accounting for the design effect (i.e. without weighting).

Reduction of the dimension of the dataset by Multiple Correspondance analysis – Selection of the number of axes to retain in analysis

Table S2. Inertia and Chi-square decomposition

| Singular value | Principal Inertia | Chi-2 | % | Cumulative % | Loss of inertia | Benzecri adjusted  inertia | | 2 4 6 8 10 |
| --- | --- | --- | --- | --- | --- | --- | --- | --- |
|  |  |  |  |  |  | % | Cum % | ----+----+----+----+--- |
| 0,371 | 0,137 | 2854,9 | 6,90 | 6,90 | 4,39 | 13,73 | 13,73 | ***************** |
| 0,363 | 0,132 | 2735,8 | 6,61 | 13,5 | 22,18 | 13,03 | 26,76 | ***************** |
| 0,329 | 0,108 | 2241,1 | 5,41 | 18,92 | 6,71 | 10,18 | 36,94 | ************** |
| 0,318 | 0,101 | 2099,5 | 5,07 | 23,99 | 4,54 | 9,37 | 46,31 | ************* |
| 0,311 | 0,096 | 2007,3 | 4,85 | 28,84 | 13,05 | 8,86 | 55,16 | ************ |
| 0,292 | 0,085 | 1776 | 4,29 | 33,13 | 5,15 | 7,57 | 62,73 | *********** |
| 0,285 | 0,081 | 1691 | 4,08 | 37,21 | 6,25 | 7,10 | 69,83 | ********** |
| 0,277 | 0,076 | 1589 | 3,84 | 41,05 | 4,63 | 6,54 | 76,38 | ********** |
| 0,270 | 0,073 | 1518,4 | 3,67 | 44,72 | 1,94 | 6,16 | 82,54 | ********* |
| 0,268 | 0,071 | 1488,5 | 3,60 | 48,31 | 3,15 | 6,00 | 88,54 | ********* |
| 0,264 | 0,069 | 1446,8 | 3,49 | 51,81 | 1,16 | 5,78 | 94,32 | ********* |
| 0,262 | 0,068 | 1428 | 3,45 | 55,26 |  | 5,68 | 100,00 | ********* |
| 0,258 | 0,066 | 1380,7 | 3,34 | 58,59 |  |  |  | ******** |
| 0,256 | 0,065 | 1360,6 | 3,29 | 61,88 |  |  |  | ******** |
| 0,254 | 0,064 | 1341,5 | 3,24 | 65,12 |  |  |  | ******** |
| 0,252 | 0,063 | 1324,3 | 3,20 | 68,32 |  |  |  | ******** |
| 0,251 | 0,063 | 1306,1 | 3,15 | 71,47 |  |  |  | ******** |

Note. The number of axes was determined by a marked reduction of the inertia if the cumulative percent of inertia reached is large enough

Hierarchical cluster analysis (HCA)

Figure S1. Graphical determination of the optimal number of classes/profiles using pseudo T² criteria

| Pseudo T² |
| --- |
| 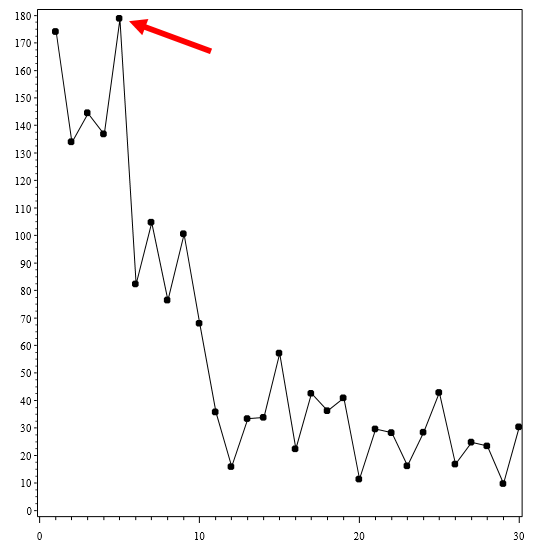 |
| Number of classes |
|  |

Note. The number of classes is determined by a slope discontinuity. Other criteria were used, such as the R-squared, semi-partial R-squared, and cubic classification. They were consistent in identifying 5 classes.

Figure S2. Graphical representation of the 5 subgroups/profiles by projection on the first 4 axes

| 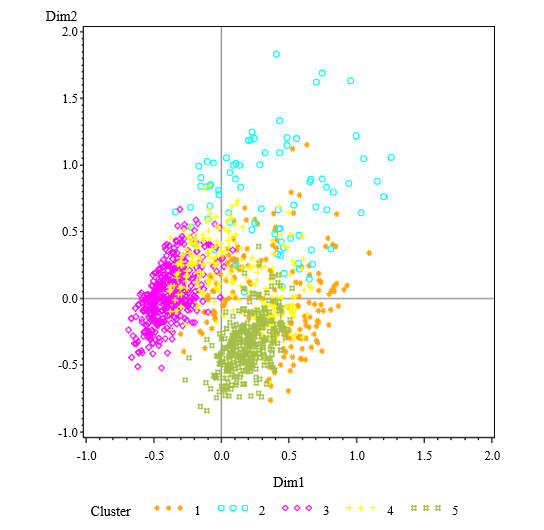 | 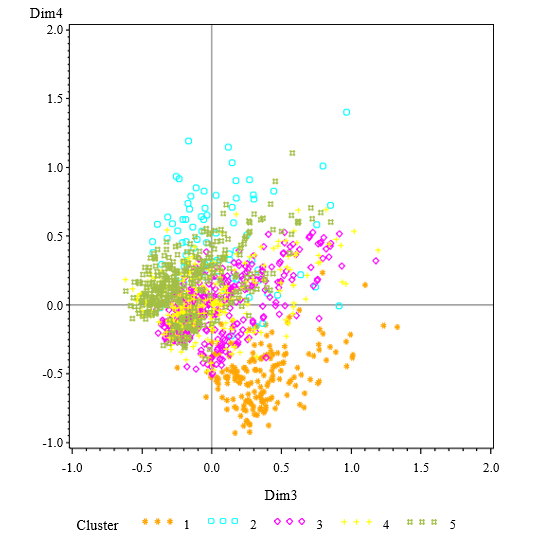 |
| --- | --- |
